# One-Year Evolution of Macrovascular and Microvascular Parameters After Endovascular Revascularization in Diabetic Foot Ulcers: A Prospective Multicenter Study

**DOI:** 10.3390/jcm15187278

**Published:** 2026-09-19

**Authors:** Israel Anguiano-Marcos, Sebastián Flores-Escobar, Yolanda García-Álvarez, Aroa Tardáguila-García, José Luis Lázaro-Martínez, Francisco Javier Álvaro-Afonso

**Affiliations:** 1 Diabetic Foot Unit, Clínica Universitaria de Podología, Facultad de Enfermería, Fisioterapia y Podología, Universidad Complutense de Madrid, 28040 Madrid, Spain; israngui@ucm.es (I.A.-M.); ygarci01@ucm.es (Y.G.-Á.); aroa.tardaguila@ucm.es (A.T.-G.); diabetes@ucm.es (J.L.L.-M.); alvaro@ucm.es (F.J.Á.-A.); 2 Instituto de Investigación Sanitaria del Hospital Clínico San Carlos (IdISSC), 28040 Madrid, Spain

**Keywords:** diabetic foot, ischemia, vascular assessment, peripheral arterial disease, wound healing

## Abstract

**Background**: This study aimed to characterize the one-year longitudinal evolution of macrovascular and microvascular parameters following endovascular revascularization in patients with ischemic or neuroischemic diabetic foot ulcers (DFUs). Associations between early post-revascularization vascular parameters and wound-healing outcomes were evaluated as secondary exploratory analyses. **Methods**: A prospective multicenter longitudinal study included 27 ischemic or neuroischemic DFUs undergoing endovascular revascularization. Patients were followed for 12 months with serial assessments of ankle systolic pressure (AP), toe systolic pressure (TP), ankle-brachial pressure index (ABPI), toe-brachial pressure index (TBPI), transcutaneous oxygen pressure (TcPO_2_), and skin perfusion pressure (SPP). Secondary exploratory analyses evaluated associations between early vascular parameters and wound-healing outcomes. **Results:** Twenty-six ulcers (96.3%) achieved complete healing, with no major amputations or deaths during follow-up. AP, TP, ABPI, and TBPI improved significantly over 12 months (all *p* < 0.001), whereas TcPO_2_ showed a significant overall longitudinal effect (*p* = 0.010) despite greater variability, and SPP remained unchanged (*p* = 0.177). Exploratory secondary analyses suggested that higher AP at four weeks after revascularization was associated with shorter healing time (ρ = −0.451, *p* = 0.024), while higher TcPO_2_ values were observed in ulcers with early wound healing (*p* = 0.040). **Conclusions**: Macrovascular and microvascular parameters exhibit distinct recovery patterns following endovascular revascularization. Longitudinal assessment of both vascular compartments may provide complementary information for monitoring vascular recovery. The exploratory associations observed between early vascular parameters and wound-healing outcomes warrant confirmation in larger adequately powered studies.

## 1. Introduction

Diabetic foot ulcer (DFU) is one of the most frequent and devastating complications of diabetes mellitus and is a major cause of morbidity, lower-extremity amputation, and mortality worldwide. The lifetime incidence of DFU ranges from 19% to 34%, and recurrence is still high despite improvements in prevention and treatment strategies [1]. Moreover, patients with a history of DFU have significantly higher risk of death, and mortality rates at 5 years after major amputation are as high as 70% [2,3]. Macrovascular disease plays a central role in tissue ischemia, but increasing evidence suggests that microvascular dysfunction also contributes significantly to impaired healing and adverse outcomes among people with diabetes [4]. Furthermore, the neuroischemic type has become the most common clinical presentation of DFU, which emphasizes the importance of comprehensive vascular assessment in this context [1].

In addition to vascular and neuropathic impairment, altered foot biomechanics and persistently elevated plantar pressure are important contributors to DFU development, impaired healing, and recurrence. Therefore, effective pressure redistribution represents an essential component of DFU management. In appropriately selected patients with chronic plantar ulcers, minimally invasive metatarsal osteotomies have been investigated as a surgical off-loading strategy to reduce plantar pressure, promote ulcer healing, and prevent recurrence [5,6].

Endovascular revascularization is a cornerstone in the management of chronic limb-threatening ischemia (CLTI) and DFUs. When feasible, angiosome-oriented revascularization strategies have been associated with improved wound healing and limb-salvage outcomes and may reduce the costs of healthcare related to major amputations [7]. Nevertheless, predicting wound healing after revascularization is challenging, and clinicians rely on several non-invasive bedside vascular tests to estimate healing potential and guide treatment decisions.

A wide range of non-invasive vascular tests have been investigated as prognostic tools for wound healing and limb outcomes in patients with DFU. Examples include the ankle systolic pressure (AP), ankle-brachial pressure index (ABPI), toe systolic pressure (TP), toe-brachial pressure index (TBPI), transcutaneous oxygen pressure (TcPO_2_), and skin perfusion pressure (SPP) [8]. However, despite their widespread clinical use, no single vascular test has demonstrated sufficient prognostic accuracy when used in isolation. More recently, in a prospective multicenter study, measurements of TcPO_2_ and TP within 4 weeks after endovascular revascularization demonstrated the highest prognostic performance for predicting wound healing. These findings demonstrate the importance of microvascular assessment and suggest that microcirculatory parameters may provide clinically relevant information beyond traditional macrovascular measurements [9].

The most recent international intersocietal guidelines recommend the use of TP and TcPO_2_ measurements to estimate the probability of healing and the risk of amputation for people with DFU [10]. An updated systematic review has emphasized the growing importance of microvascular assessment and suggests that TcPO_2_ and SPP provide clinically meaningful prognostic information. Nevertheless, the overall predictive performance of currently available non-invasive vascular tests is moderate, and there has been considerable heterogeneity between published studies [8,11].

Previous studies have demonstrated the prognostic value of TcPO_2_ and TP following endovascular revascularization. However, vascular recovery after endovascular revascularization is a dynamic process, and little is known about the long-term longitudinal evolution of macrovascular and microvascular parameters and their relationship with wound-healing dynamics. Most studies have focused on baseline measurements or short-term post-procedural changes, and there are important questions regarding the temporal behavior of vascular parameters after revascularization. Thus, longitudinal studies are needed to evaluate vascular changes over time and their potential prognostic value for wound-healing outcomes after revascularization. The main aim of this study was to characterize the longitudinal changes in macrovascular and microvascular parameters within 12 months after endovascular revascularization among patients with ischemic or neuroischemic DFU. The secondary exploratory objectives were to assess the association between early post-revascularization vascular parameters and wound-healing time, and to compare early vascular parameters between early-healing and delayed-healing ulcers.

## 2. Materials and Methods

### 2.1. Study Design

This prospective longitudinal observational study examined patients with ischemic or neuroischemic DFU who underwent endovascular revascularization for CLTI. The study is part of an ongoing cohort with a planned sample size of 120 patients, as specified in the study protocol approved by the Ethics Committee. The present analysis included 27 DFUs from 25 consecutive patients who had completed the scheduled 12-month follow-up at the time of the present analysis. Patients were enrolled between January 2022 and April 2025. The DFU was considered the unit of analysis, and two patients contributed two ulcers each. In both patients, the two ulcers were located on different lower limbs, and vascular measurements were obtained separately for each affected limb. Therefore, ulcer-level characteristics, vascular measurements, and healing outcomes were analyzed separately for each DFU. For patient-level baseline characteristics, each patient was counted once using data from the first study inclusion. Serial macrovascular and microvascular assessments were performed before revascularization (Visit 0), during follow-up at 4 weeks (Visit 1), and then every 8 weeks during follow-up after the revascularization procedure (Visit 2: 12 weeks; Visit 3: 20 weeks; Visit 4: 28 weeks; Visit 5: 36 weeks; Visit 6: 44 weeks; Visit 7: 52 weeks). The study received approval from the Ethics Committee at Hospital Clínico San Carlos, Madrid, Spain (C.P.-C.I. 21/596-E, date of approval: 22 September 2021). All participating patients provided written informed consent prior to participation. In addition, the authors affirmed adherence to the ethical principles of the Declaration of Helsinki [12].

### 2.2. Participants

Patients were eligible for inclusion if they were aged ≥18 years, had type 1 or type 2 diabetes, and presented with an ischemic or neuroischemic DFU requiring endovascular revascularization. CLTI was diagnosed according to current international recommendations based on clinical assessment and non-invasive vascular testing. Eligible patients presented at least one of the following hemodynamic criteria: AP < 50 mmHg, TP < 30 mmHg, TcPO_2_ < 30 mmHg, or ABPI < 0.40. Ulcers were classified according to the University of Texas Wound Classification System, and only ischemic or neuroischemic ulcers corresponding to stages IC, IIC, IIIC, ID, IID, or IIID were included. Patients were excluded if they had lower-limb lymphedema; previous major contralateral amputation; wounds whose size or anatomical location prevented reliable placement of the probes or cuffs required for non-invasive vascular assessments; an estimated life expectancy of less than 6 months; active malignancy under treatment; a non-salvageable limb, defined as a limb considered by the Vascular Surgery team to require primary major amputation rather than revascularization based on the overall clinical and anatomical assessment; inability to ambulate independently, consistent with the clinical criteria applied by the Vascular Surgery team when assessing candidacy for revascularization; or physical or cognitive impairment preventing participation in the study or completion of follow-up assessments.

### 2.3. Procedures

All consecutive patients who met the inclusion criteria were recruited after providing written informed consent at one of the research centers (Diabetic Foot Unit, Podiatric Clinic, Complutense University of Madrid, Spain; Vascular Surgery Department, Hospital Fundación Alcorcón, Madrid, Spain; Vascular Surgery Department, Hospital Universitario Ramón y Cajal, Madrid, Spain; Vascular Surgery Department, Hospital Quirón Pozuelo, Madrid, Spain; Vascular Surgery Department, Hospital Quirón La Luz, Madrid, Spain). Baseline demographic and clinical characteristics were recorded, including age; sex; diabetes type and duration; glycated hemoglobin (HbA1c); comorbidities; ulcer characteristics, including University of Texas classification and anatomical location; and previous lower-extremity interventions.

Detailed procedural characteristics, including the number and specific vessels treated, angiosome-directed revascularization, pedal arch status, and the use of drug-eluting devices, were not prospectively collected in a standardized manner for the purposes of the present study and were therefore not included in the statistical analyses.

Noninvasive tests were performed using the PeriFlux 6000 System (Perimed AB, Järfälla, Sweden). Measurements were obtained after at least 10 min of supine rest in a temperature-controlled room (20–23 °C) at each study visit. For peripheral pressure measurements, three consecutive determinations were obtained at each measurement site using the PeriFlux 6000 system, and the mean value was used for analysis. All assessments were performed by clinicians with experience in vascular examination. Throughout follow-up, all patients received standard multidisciplinary diabetic foot care. Care included regular wound debridement, culture-guided antibiotic therapy and surgical debridement for soft tissue or bone infections, moisture-balanced dressings, customized pressure offloading, and vascular surveillance according to international guidelines.

### 2.4. Outcome Measures

The primary outcome was the longitudinal change in macrovascular parameters (AP, TP, ABPI, and TBPI) and microvascular parameters (TcPO_2_ and SPP) during the 12 months of follow-up after endovascular revascularization. The secondary clinical outcomes were complete wound healing, time to healing, and major amputation. Associations between early post-revascularization vascular parameters and wound-healing outcomes were evaluated separately as secondary exploratory analyses. Wound healing was defined as complete epithelialization of the ulcer without drainage that was maintained for at least 2 weeks [13].

To minimize potential assessment bias, the achievement of complete healing was independently evaluated and confirmed by a second experienced clinician who was blinded to the patient’s longitudinal macrovascular and microvascular measurement results. Time to healing was defined as the interval between endovascular revascularization and complete wound healing expressed in weeks. Major amputation was defined as any amputation performed above the ankle during follow-up [13].

### 2.5. Statistical Analysis

All statistical analyses were performed using IBM SPSS Statistics version 29.0 (IBM Corp., Armonk, NY, USA). Categorical variables were expressed as frequencies and percentages, while continuous variables were reported as the mean ± standard deviation (SD) or median and interquartile range (IQR). A *p*-value < 0.05 was considered statistically significant.

Repeated-measures analysis of variance (ANOVA) was performed to evaluate longitudinal changes in vascular parameters after endovascular revascularization. Analyses were conducted using complete cases for each vascular parameter. Normality of standardized residuals was assessed using the Shapiro–Wilk test. No systematic departures from normality were observed, although isolated deviations were detected at individual assessment points for some vascular parameters. Sphericity was formally assessed using Mauchly’s test, and the Greenhouse–Geisser correction was applied when this assumption was violated. Post hoc comparisons between each follow-up visit and baseline (Visit 0) were performed using Bonferroni correction for multiple comparisons. Effect sizes were reported using partial eta squared (*η*p^2^). No global multiplicity correction was applied across the secondary exploratory analyses; therefore, the corresponding *p*-values should be considered nominal.

An a priori sample size calculation was performed at the study design stage using R software version 3.6.2 (pwr package, R Foundation for Statistical Computing, Vienna, Austria). Assuming a statistical power of 80%, a type II error (β) of 20%, an alpha level of 0.05, and a 95% confidence level, the minimum required sample size was estimated at 120 participants for the originally planned logistic regression analyses. Recruitment is ongoing, and 27 DFUs from 25 participants have been included in the study to date. Therefore, analyses based on the currently available cohort should be considered exploratory and interpreted with caution until the prespecified sample size is reached.

The association between vascular parameters measured 4 weeks after revascularization (Visit 1) and wound-healing time was assessed using Spearman’s rank correlation coefficient. For exploratory analyses, DFUs were divided into an early healing group (≤10 weeks) and a delayed-healing group (>10 weeks). The 10-week threshold was selected because it approximated the median healing time of the study cohort and was considered an exploratory rather than a prespecified clinical cutoff.

Continuous variables were compared using the independent-samples Student’s *t*-test for normally distributed variables and the Mann–Whitney *U* test for non-normally distributed variables.

To assess the potential influence of within-patient clustering, a sensitivity analysis was performed including only one DFU per patient. For the two patients who contributed the two DFUs, only the first study inclusion was retained, resulting in a sensitivity cohort of 25 DFUs from 25 patients. The primary longitudinal analyses were repeated in this cohort.

## 3. Results

The final study cohort comprised 25 patients with 27 ischemic or neuroischemic DFUs who underwent endovascular revascularization and were followed for 12 months. The cohort was predominantly male (92.0%) and had a mean age of 73.4 ± 8.5 years. Almost all patients had type 2 diabetes (96.0%), and the mean disease duration was 24.0 ± 12.7 years. Previous DFU had occurred in 84.0% of patients, and one-third had previously undergone an amputation (36.0%). Approximately half of the patients had never smoked (48.0%). Baseline demographic, clinical and pharmacological patient-level characteristics are summarized in Table 1. According to the University of Texas Wound Classification System, 9 DFUs (33.3%) were classified as 1C, 1 (3.7%) as 1D, 3 (11.1%) as 2C, 1 (3.7%) as 2D, 1 (3.7%) as 3C, and 12 (44.4%) as 3D. Most ulcers were located in the forefoot (85.2%), while 7.4% were located in the midfoot and 7.4% in the hindfoot. The mean ulcer duration before study inclusion was 10.1 ± 13.8 weeks. During follow-up, 13 DFUs (48.1%) required a surgical procedure. Baseline ulcer-level and vascular characteristics are summarized in Table 2.

### 3.1. Longitudinal Changes in Macrovascular and Microvascular Parameters After Endovascular Revascularization

**Figure 1:**
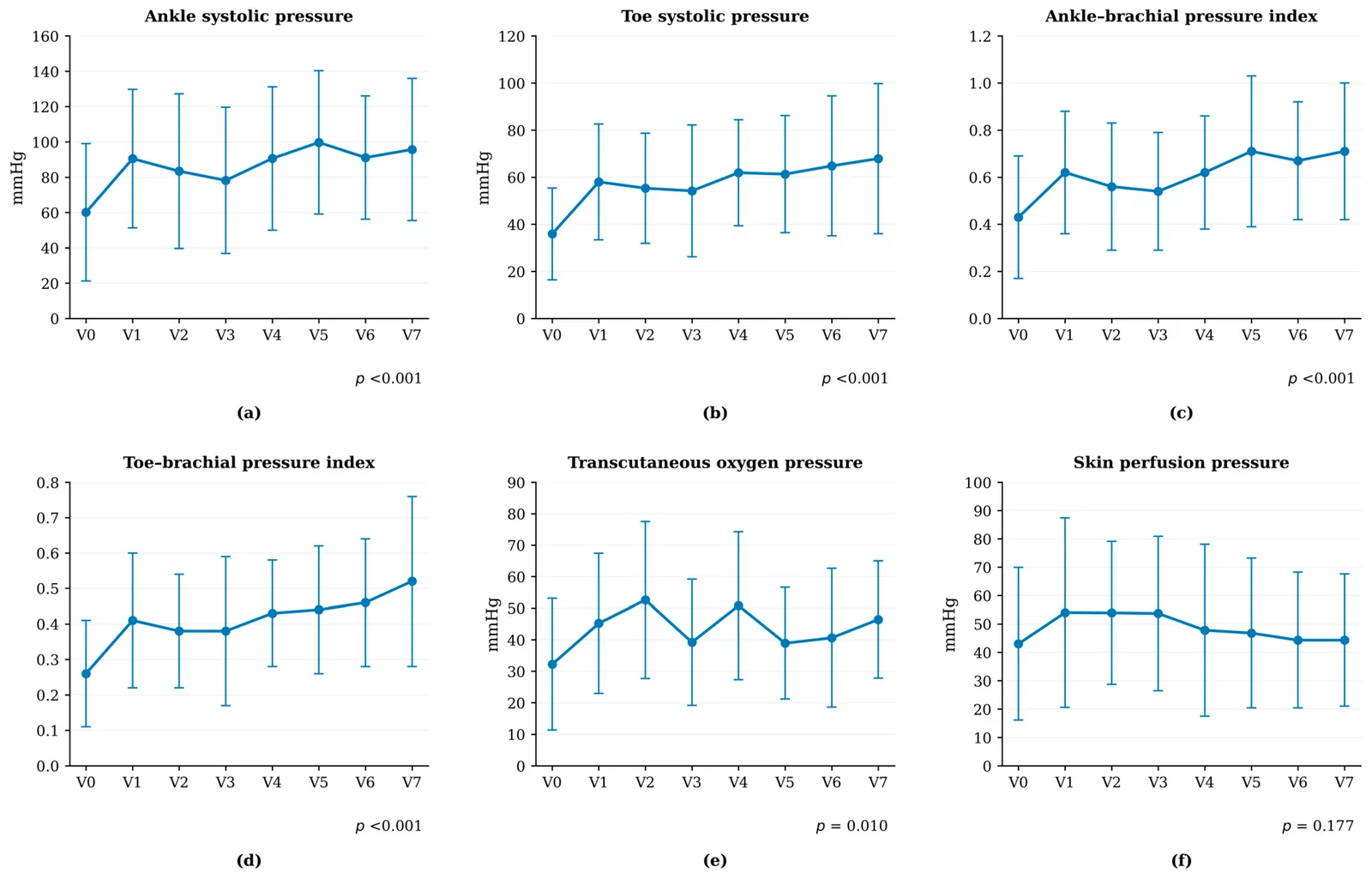
Longitudinal evolution of macrovascular and microvascular parameters (12 months). (**a**) Ankle systolic pressure; (**b**) toe systolic pressure; (**c**) ankle-brachial pressure index; (**d**) toe-brachial pressure index; (**e**) transcutaneous oxygen pressure; (**f**) skin perfusion pressure. Values presented as mean ± SD.

Table 3 and Figure 1 summarize the longitudinal changes in macrovascular and microvascular parameters throughout the 12-month follow-up after endovascular revascularization. During follow-up, 26 of 27 ulcers (96.3%) completely healed. No major amputations were performed during the 12-month follow-up. Among patients who reached the 12-month follow-up and were included in the final analytical cohort, no deaths were recorded during the follow-up period. Longitudinal changes in macrovascular and microvascular parameters are presented in Table 3 and Figure 1.

Vascular parameters showed significant longitudinal improvements after revascularization. AP increased from 60.1 ± 38.9 mmHg at baseline to 95.7 ± 40.3 mmHg at week 52 (*p* < 0.001, ηp^2^ = 0.158). Similarly, TP increased from 35.9 ± 19.5 mmHg at baseline to 67.9 ± 31.9 mmHg at week 52 (*p* < 0.001, ηp^2^ = 0.218). Both ABPI and TBPI showed significant improvements over time. ABPI increased from 0.43 ± 0.26 at baseline to 0.71 ± 0.29 at week 52 (*p* < 0.001, ηp^2^ = 0.212), while TBPI increased from 0.26 ± 0.15 to 0.52 ± 0.24 (*p* < 0.001, ηp^2^ = 0.268). Among the evaluated vascular parameters, TBPI showed the largest effect size (ηp^2^ = 0.268), followed by TP (ηp^2^ = 0.218) and ABPI (ηp^2^ = 0.212). These effect sizes quantify the magnitude of the within-subject temporal effect observed in the present dataset and should not be interpreted as evidence of clinical superiority of one vascular parameter over another.

TcPO_2_ also showed a significant overall longitudinal effect (*F* = 2.751, *p* = 0.010, ηp^2^ = 0.121) and increased from 32.2 ± 20.9 mmHg at baseline to 46.4 ± 18.6 mmHg at week 52. However, none of the pairwise comparisons had statistically significant results after Bonferroni correction, indicating substantial inter-individual variability during follow-up. In contrast, SPP remained stable throughout follow-up, and no significant longitudinal changes were observed (*p* = 0.177).

A sensitivity analysis including only one DFU per patient (25 DFUs from 25 patients) yielded results consistent with the primary analysis. Significant longitudinal effects remained for AP (*p* = 0.003), TP (*p* = 0.001), ABPI (*p* < 0.001), TBPI (*p* < 0.001), and TcPO_2_ (*p* = 0.016), whereas SPP remained non-significant (*p* = 0.404). Thus, exclusion of the two additional DFUs did not materially alter the primary longitudinal findings.

### 3.2. Association Between Early Vascular Status and Wound-Healing Time

Table 4 shows the association between early post-revascularization vascular parameters and wound-healing time. Healing-time data were available for 25 DFUs. The median healing time was 11.7 weeks (IQR 5.7–20.9 weeks). Among the evaluated parameters, AP at Visit 1 showed an inverse correlation with healing time (ρ = −0.451, *p* = 0.024), suggesting that higher AP values may be associated with shorter healing time. No statistically significant correlations were observed for TP, ABPI, TBPI, TcPO_2_, or SPP (*p* > 0.05). Given the multiple secondary comparisons performed, these findings should be interpreted as exploratory and hypothesis-generating.

### 3.3. Comparison of Early Vascular Parameters According to Healing Status

Table 5 shows the exploratory comparison of early post-revascularization vascular parameters between patients with early and delayed wound healing. DFUs were classified according to wound-healing status at 10 weeks as early healers (≤10 weeks, *n* = 11) and delayed healers (>10 weeks, *n* = 14). No statistically significant differences were observed for AP, TP, ABPI, TBPI, or SPP. However, TcPO_2_ measured at Visit 1 (4 weeks after revascularization) was higher for ulcers that healed within 10 weeks than those requiring more than 10 weeks for healing (42.5 ± 19.4 vs. 25.0 ± 20.5 mmHg, *p* = 0.040). Given the exploratory nature of this analysis and the multiple secondary comparisons performed, this finding should be interpreted as hypothesis-generating rather than confirmatory.

## 4. Discussion

In this prospective longitudinal study, we characterized the temporal evolution of macrovascular and microvascular parameters during the first year after endovascular revascularization among patients with ischemic or neuroischemic DFUs. The study provides longitudinal evidence that vascular parameters exhibit dynamic changes during follow-up after endovascular revascularization, with macrovascular and microvascular parameters showing distinct temporal trajectories. Macrovascular parameters showed sustained improvement after revascularization, while microvascular parameters exhibited a more heterogeneous pattern of recovery. This suggests that restoration of arterial inflow does not necessarily translate into immediate normalization of tissue perfusion. Furthermore, secondary exploratory analyses suggested associations between early post-revascularization vascular parameters and wound-healing outcomes, supporting the potential complementary rather than interchangeable information provided by macrovascular and microvascular measurements during follow-up. These findings provide new insights into the temporal behavior of vascular parameters after revascularization and support a more comprehensive approach to vascular assessment for patients with ischemic or neuroischemic DFUs.

Previous studies have primarily evaluated vascular status at isolated time points or focused on the prognostic performance of individual vascular tests. In contrast, the present study characterized vascular recovery as a dynamic process involving distinct temporal responses of the macrovascular and microvascular compartments. Macrovascular parameters showed a relatively homogeneous pattern of recovery, with AP, TP, ABPI, and TBPI showing significant sustained improvements throughout the 12-month follow-up (Table 3 and Figure 1). These findings are consistent with previous reports describing restoration and maintenance of arterial inflow after revascularization [10,14].

In contrast, TcPO_2_ exhibited a more heterogeneous temporal trajectory and increased from 32.2 ± 20.9 mmHg at baseline to 46.4 ± 18.6 mmHg at 52 weeks. Nevertheless, none of the pairwise comparisons indicated statistically significant results after Bonferroni correction (Table 3). This greater variability suggests that recovery of tissue oxygenation is influenced by mechanisms other than correction of large-vessel obstruction. Similar observations have been reported in previous studies. These studies showed that restoration of arterial inflow does not necessarily result in immediate normalization of tissue oxygenation because persistent microvascular dysfunction may continue to limit tissue perfusion after revascularization [4,14].

These findings are consistent with those reported by Schönborn et al., who prospectively evaluated microcirculatory changes after endovascular revascularization in 41 patients with diabetic foot syndrome. In their study, TcPO_2_ increased from 17.3 ± 12.1 mmHg before revascularization to 44.9 ± 14.8 mmHg at 12 months, with a progressive improvement observed during follow-up. Although baseline TcPO_2_ values and assessment intervals differed between studies, both investigations showed an overall improvement in tissue oxygenation during the 12 months following endovascular revascularization. Notably, Schönborn et al. also evaluated laser Doppler flowmetry and circulating angiogenic factors, whereas the present study integrates TcPO_2_ and SPP with serial macrovascular measurements. Taken together, these findings support the concept that microvascular recovery after restoration of arterial inflow is a dynamic process and may not parallel changes in macrovascular perfusion [15].

The different temporal behavior observed between vascular compartments is consistent with the concept that restoration of arterial inflow is only one component of tissue recovery after revascularization. Although arterial patency can be re-established immediately after the procedure, recovery of tissue oxygenation may reflect progressive improvement in microvascular function and other biological processes that are involved in tissue repair rather than the immediate restoration of arterial blood flow alone [16,17]. Thus, restoration of macrovascular perfusion does not necessarily translate into immediate normalization of tissue oxygenation, particularly in individuals with diabetes, for which persistent microvascular dysfunction may continue to impair oxygen delivery despite successful restoration of arterial blood flow [4,18,19]. These observations are consistent with growing evidence that diabetes-related microvascular dysfunction is a distinct pathological process that cannot be fully explained by the severity of large-vessel disease alone [16,17].

Interestingly, TBPI showed the largest effect size for longitudinal change among the vascular parameters evaluated. This finding indicates a comparatively larger within-subject temporal effect in the present dataset but does not establish greater clinical usefulness, diagnostic accuracy, prognostic value, or superiority over the other vascular parameters. Toe-based measurements may nevertheless offer methodological advantages in patients with diabetes because they are generally less affected by medial arterial calcification than ankle-derived indices [20]. Therefore, our findings are consistent with previous studies suggesting that assessments based on toe pressure may provide a more accurate evaluation of distal perfusion and may be particularly useful for monitoring vascular status after endovascular revascularization [10]. Nevertheless, the clinical implications of the greater responsiveness observed for TBPI should be confirmed in larger prospective studies.

The relationship between early vascular status and wound healing also supports the concept that macrovascular and microvascular assessments provide complementary information during post-revascularization follow-up rather than being competing approaches. AP measured 4 weeks after revascularization was associated with subsequent healing time, and higher TcPO_2_ values were associated with early healing. These findings suggest that macrovascular parameters primarily reflect restoration of arterial inflow, and TcPO_2_ may provide additional information regarding tissue oxygenation during the early biological phases of wound healing [9,19,21].

These observations complement the findings of our previous multicenter prospective study, which identified TcPO_2_ and TP as the strongest predictors of wound-healing after endovascular revascularization [9]. Importantly, both studies were designed to answer different clinical questions. The previous study evaluated the prognostic performance of vascular tests at an early time point after revascularization, while the present investigation describes their temporal evolution throughout the first year after revascularization. Together, the findings support a longitudinal approach to vascular assessment in which both the prognostic performance and the temporal behavior of vascular parameters contribute complementary information for clinical decision-making.

The high proportion of complete wound healing observed in the present cohort (96.3%), together with the absence of major amputations during follow-up, should be interpreted in the context of the specialized multidisciplinary management provided throughout the study. Following endovascular revascularization, patients underwent close follow-up in specialized diabetic foot units and received comprehensive wound care, including regular debridement, infection management, appropriate offloading, and vascular surveillance. When clinically indicated, surgical procedures were performed as part of the limb-salvage strategy; however, the specific type of procedure was not systematically categorized in the study dataset. Therefore, the favorable clinical outcomes observed in this cohort should be interpreted in the context of both restoration of arterial perfusion and the comprehensive multidisciplinary management provided throughout follow-up; however, given the observational design and absence of a comparator group, the contribution of individual components of care to these outcomes cannot be determined.

Among patients who reached the 12-month follow-up and were included in the final analytical cohort, no deaths were recorded during the follow-up period. Although CLTI is generally associated with substantial one-year mortality [3,14], this finding should be interpreted cautiously given the limited sample size, the absence of a comparator group, and the characteristics of the final analytical cohort.

In addition, the predominance of male participants (92.0%) may limit the generalizability of these findings, particularly to female patients.

The present findings have several important clinical implications. The results support a longitudinal approach to vascular assessment after endovascular revascularization as vascular recovery extends beyond the immediate post-procedural period. Rather than relying on a single post-revascularization evaluation, serial vascular assessment may provide a more comprehensive understanding of the recovery process and help to identify patients whose restoration of arterial inflow is not accompanied by an equivalent recovery of tissue oxygenation. Thus, closer surveillance and more individualized wound management are warranted. 

The findings also reinforce the complementary value of combining macrovascular and microvascular assessment during follow-up. Macrovascular parameters provide objective evidence of sustained restoration of arterial perfusion, while microvascular assessment gives additional insight into tissue oxygenation and the biological response of the wound to revascularization. Thus, integrating both vascular compartments into routine clinical practice may improve the evaluation of post-revascularization recovery and facilitate more individualized clinical decision-making after endovascular revascularization [9,10,11,22].

To our knowledge, this prospective longitudinal study is among the first to characterize both macrovascular and microvascular recovery over 12 months after endovascular revascularization among patients with ischemic or neuroischemic DFUs. Repeated vascular assessments performed at predefined intervals enabled detailed characterization of the temporal evolution of lower-limb perfusion and provided information that has been scarcely reported in previous studies. Nevertheless, several limitations should be acknowledged.

An important limitation of the present analysis is that the prespecified sample size has not yet been reached. The a priori sample size calculation estimated that at least 120 participants would be required, whereas 27 DFUs have been included to date. Therefore, the findings of the present analysis, particularly those derived from secondary analyses, should be interpreted with caution. Non-significant findings should not be considered evidence of absence of an association, as limited statistical power and type II error cannot be excluded. Further recruitment is ongoing to achieve the prespecified sample size.

The DFU was considered the unit of analysis, and two patients contributed two ulcers each. Although the two DFUs contributed by each of these patients were located on different limbs and had independent limb-specific vascular measurements, some degree of within-patient correlation due to shared patient-level characteristics cannot be excluded. However, a sensitivity analysis including only one DFU per patient yielded results consistent with the primary longitudinal analysis, suggesting that within-patient clustering did not materially affect the main findings.

The observational design precludes causal inference.

Another limitation is the absence of a comparator group, such as patients with non-ischemic DFUs not requiring revascularization. Consequently, the observed longitudinal changes cannot be attributed exclusively to the revascularization procedure, as other factors related to wound healing, multidisciplinary care, and the natural evolution of vascular parameters may also have contributed. The lack of a comparator group also limits our ability to determine whether the temporal vascular patterns observed are specific to ischemic or neuroischemic DFUs undergoing revascularization. Future controlled prospective studies including appropriate comparator groups are needed to clarify these relationships.

Detailed procedural and anatomical characteristics of endovascular revascularization, including the number and specific vessels treated, angiosome-directed revascularization, pedal arch status, and the use of drug-eluting devices, were not prospectively collected in a standardized manner and therefore could not be evaluated in the present study. These factors may influence post-revascularization perfusion and wound-healing outcomes and represent potential sources of residual confounding. Future studies should incorporate detailed anatomical and procedural data to determine their influence on longitudinal macrovascular and microvascular recovery.

Despite these limitations, the present study provides longitudinal evidence on the evolution of macrovascular and microvascular perfusion during the first year after endovascular revascularization. The distinct temporal patterns observed between vascular compartments support the potential value of a complementary approach combining macrovascular and microvascular assessment during follow-up of patients with ischemic or neuroischemic DFUs.

## 5. Conclusions

This prospective longitudinal study showed that macrovascular and microvascular parameters followed distinct temporal trajectories during the first year after endovascular revascularization in patients with ischemic or neuroischemic DFUs. Macrovascular parameters showed sustained improvement throughout follow-up, whereas tissue oxygenation exhibited a more heterogeneous temporal pattern. These findings suggest that longitudinal assessment of macrovascular and microvascular parameters may provide complementary information when monitoring vascular status after revascularization. Associations between early vascular parameters and wound-healing outcomes were observed in secondary exploratory analyses and should be interpreted as hypothesis-generating, particularly given the limited sample size and selected study population. Further controlled prospective studies in larger and more heterogeneous cohorts are needed to confirm these findings and determine their clinical and prognostic relevance.

## Figures and Tables

**Table 1:** Baseline patient-level characteristics of the study population.

Characteristic	N = 25
Male, n (%)	23 (92.0)
Female, n (%)	2 (8.0)
Age (years), mean ± SD	73.4 ± 8.5
Type 2 diabetes, n (%)	24 (96.0)
Diabetes mellitus (years), mean ± SD	24.0 ± 12.7
HbA1c (%), median (IQR)	7.3 (7.0–8.5)
Hypertension, n (%)	23 (92.0)
Hypercholesterolemia, n (%)	22 (88.0)
Neuropathy, n (%)	18 (72.0)
Retinopathy, n (%)	6 (24.0)
Nephropathy, n (%)	5 (20.0)
Cardiopathy, n (%)	9 (36.0)
Previous DFU, n (%)	21 (84.0)
Previous amputation, n (%)	9 (36.0)
Antiplatelet treatment, n (%)	22 (88.0)
Statins, n (%)	21 (84.0)
Anticoagulants, n (%)	4 (16.0)
Antihypertensive treatment, n (%)	24 (96.0)
Oral antidiabetic drugs, n (%)	16 (64.0)
Insulin therapy, n (%)	16 (64.0)

DFU, diabetic foot ulcer; SD, standard deviation; IQR, interquartile range.

**Table 2:** Baseline ulcer-level and vascular characteristics.

Characteristic	N = 27 DFUs
**University of Texas Wound Classification System, n (%)**	
1C	9 (33.3)
1D	1 (3.7)
2C	3 (11.1)
2D	1 (3.7)
3C	1 (3.7)
3D	12 (44.4)
**Ulcer duration before study inclusion (weeks), mean ± SD**	10.1 ± 13.8
**Anatomical location, n (%)**	
Forefoot	23 (85.2)
Midfoot	2 (7.4)
Hindfoot	2 (7.4)
**Surgical procedure during follow-up, n (%)**	
Yes	13 (48.1)
No	14 (51.9)
**Baseline vascular measurements**	
AP (mmHg), median (IQR)	53.0 (38.0–76.0)
TP (mmHg), mean ± SD	35.9 ± 19.5
ABPI, median (IQR)	0.35 (0.27–0.57)
TBPI, median (IQR)	0.23 (0.13–0.39)
TcPO_2_ (mmHg), mean ± SD	32.2 ± 20.9
SPP (mmHg), median (IQR)	37.5 (23.3–53.3)

AP, ankle systolic pressure; TP, toe systolic pressure; ABPI, ankle-brachial pressure index; TBPI, toe-brachial pressure index; TcPO_2_, transcutaneous oxygen pressure; SPP, skin perfusion pressure; SD, standard deviation; IQR, interquartile range. Ulcer-level characteristics and baseline vascular measurements were calculated for all 27 DFUs according to their corresponding baseline assessment. The specific type of surgical procedure was not systematically categorized in the study dataset.

**Table 3:** Longitudinal changes in vascular parameters after endovascular revascularization.

Vascular Parameter	Visit 0 (Baseline)	Visit 1 (4 Weeks)	Visit 2 (12 Weeks)	Visit 3 (20 Weeks)	Visit 4 (28 Weeks)	Visit 5 (36 Weeks)	Visit 6 (44 Weeks)	Visit 7 (52 Weeks)	*p*-Value	Effect Size
**AP (mmHg)**	60.1 ± 38.9	90.5 ± 39.2	83.4 ± 43.8	78.2 ± 41.4	90.6 ± 40.6	99.7 ± 40.6	91.1 ± 34.9	95.7 ± 40.3	<0.001 *	0.158
**TP (mmHg)**	35.9 ± 19.5	58.0 ± 24.6	55.3 ± 23.4	54.2 ± 28.0	61.9 ± 22.5	61.3 ± 24.9	64.8 ± 29.7	67.9 ± 31.9	<0.001 *	0.218
**ABPI**	0.43 ± 0.26	0.62 ± 0.26	0.56 ± 0.27	0.54 ± 0.25	0.62 ± 0.24	0.71 ± 0.32	0.67 ± 0.25	0.71 ± 0.29	<0.001 *	0.212
**TBPI**	0.26 ± 0.15	0.41 ± 0.19	0.38 ± 0.16	0.38 ± 0.21	0.43 ± 0.15	0.44 ± 0.18	0.46 ± 0.18	0.52 ± 0.24	<0.001 *	0.268
**TcPO_2_ (mmHg)**	32.2 ± 20.9	45.2 ± 22.2	52.6 ± 24.9	39.2 ± 20.0	50.8 ± 23.5	38.9 ± 17.7	40.6 ± 22.0	46.4 ± 18.6	0.010 *	0.121
**SPP (mmHg)**	43.0 ± 26.9	54.0 ± 33.4	53.9 ± 25.2	53.7 ± 27.2	47.8 ± 30.3	46.8 ± 26.4	44.3 ± 23.9	44.3 ± 23.3	0.177	0.091

AP, ankle systolic pressure; TP, toe systolic pressure; ABPI, ankle-brachial pressure index; TBPI, toe-brachial pressure index; TcPO_2_, transcutaneous oxygen pressure; SPP, skin perfusion pressure. *p*-values obtained from repeated-measures ANOVA. Effect sizes are reported as partial eta-squared (ηp^2^). Greenhouse–Geisser correction was applied when the assumption of sphericity was violated. * *p* < 0.05.

**Table 4:** Correlation between early vascular parameters and time to healing.

Vascular Parameter at 4 Weeks Post-Revascularization (Visit 1)	Spearman’s *ρ*	*p*-Value
**AP (mmHg)**	−0.451	0.024 *
**TP (mmHg)**	0.276	0.181
**ABPI**	−0.322	0.117
**TBPI**	0.308	0.135
**TcPO_2_ (mmHg)**	−0.247	0.233
**SPP (mmHg)**	−0.158	0.506

AP, ankle systolic pressure; TP, toe systolic pressure; ABPI, ankle-brachial pressure index; TBPI, toe-brachial pressure index; TcPO_2_, transcutaneous oxygen pressure; SPP, skin perfusion pressure. (−) Negative coefficients indicate shorter healing times with higher vascular parameter values. * *p* < 0.05.

**Table 5:** Comparison of early vascular parameters according to healing status.

Vascular Parameter at 4 Weeks Post-Revascularization (Visit 1)	Delayed Healing >10 Weeks (n = 14)	Early Healing ≤10 Weeks (n = 11)	*p*-Value
**AP, mmHg**	86.2 ± 44.2	99.4 ± 35.2	0.449
**TP, mmHg**	62.5 ± 24.7	51.7 ± 24.0	0.284
**ABPI**	0.60 ± 0.30	0.64 ± 0.25	0.707
**TBPI**	0.45 ± 0.21	0.34 ± 0.15	0.148
**TcPO_2_, mmHg**	25.0 ± 20.5	42.5 ± 19.4	0.040 *
**SPP, mmHg**	51.6 ± 28.5	67.4 ± 37.5	0.299

AP, ankle systolic pressure; TP, toe systolic pressure; ABPI, ankle-brachial pressure index; TBPI, toe-brachial pressure index; TcPO_2_, transcutaneous oxygen pressure; SPP, skin perfusion pressure. * *p* < 0.05.

## Data Availability

The data presented in this study are available on request from the corresponding author due to ethical reasons and privacy.

## Abbreviations

The following abbreviations are used in this manuscript:

DFUs	Diabetic Foot Ulcers
AP	Ankle Systolic Pressure
TP	Toe Systolic Pressure
ABPI	Ankle-Brachial Pressure Index
TBPI	Toe-Brachial Pressure Index
TcPO_2_	Transcutaneous Oxygen Pressure
SPP	Skin Perfusion Pressure
CLTI	Chronic Limb-Threatening Ischemia
HbA1c	Glycated Hemoglobin
IdISSC	Instituto de Investigación Sanitaria del Hospital Clínico San Carlos
SD	Standard Deviation
IQR	Interquartile Range
ANOVA	Analysis of Variance
ηp^2^	Partial Eta Squared
CI	Confidence Interval
